# Psychosomatic problems and countermeasures in Japanese children and adolescents

**DOI:** 10.1186/1751-0759-6-6

**Published:** 2012-03-20

**Authors:** Hidetaka Tanaka, Shigenori Terashima, Magnus P Borres, Olav Thulesius

**Affiliations:** 1 Department of Pediatrics, Osaka Medical College, Osaka, Japan; 2 Department of Psychology, Kansai University, Kansai, Japan; 3 Department of Pediatrics, Institution of Women's and Children's Health, Göteborg University, Göteborg, Sweden; 4 Department of Clinical Physiology, Faculty of Health Sciences, University Hospital, Linköping, Sweden

**Keywords:** Psychosomatic disease, Orthostatic dysregulation, Anorexia nervosa, School absenteeism, Migraine

## Abstract

In Japan there are a number of children and adolescents with emotion-related disorders including psychosomatic diseases (orthostatic dysregulation, anorexia nervosa, recurrent pains), behavior problems and school absenteeism. According to our previous report, the Japanese children had significantly higher score of physical symptoms and psychiatric complaints than did the Swedish children, and these were more strongly influenced by school-related stress than by home-related stress. To enforce countermeasures for psychosomatic problems in children, the Japanese Society of Psychosomatic Pediatrics (established in 1982) have started several new projects including multi-center psychosomatic researches and society-based activities. In this article, we present an outline of our study on mental health in Japanese children in comparison with Swedish children. Countermeasures including clinical guidelines for child psychosomatic diseases are reviewed and discussed.

## Major psychosomatic problems in Japanese children and adolescents

From the 1990s to the new millennium Japan has been witnessing a considerable rise in the number of children presenting psychosomatic disorders [1]. The main symptoms in these children include headache, abdominal pain, poor early rising, nausea, fatigue, etc., and children having two or more symptoms were reported to range about 20-30% [2]. Pupils who come to school infirmaries with physical symptoms related to emotional stress has increased to more than 15% per a week [3,4]. On October 19, 1999, the research team of the Ministry of Health, Labor and Welfare performed the national survey and indicated that 8.47% children aged 10-15 years old were diagnosed with psychosomatic diseases in patients. Among these, orthostatic dysregulation (OD), the major psychosomatic disease in Japanese children, amounted up to 71%. Severe OD is considered to be strongly associated with school stress and nearly half of them develop school absenteeism. Chronic headache is also a typical psychosomatic disease, strongly associated with school absenteeism. In consistent with these, the number of school absentee amounted to 114 971 (0.31% for elementary school, 2.62% for junior high school, total 1.14%) in 2010.

## Are psychosomatic problems common in Japanese adolescents?

There are several reports on a survey of physical and mental health in children and adolescents in other countries [5,6], These studies described prevalence data of physical symptoms such as headache [7,8], or abdominal pain [9], but methods employed in these studies are different, and difficult for international comparison. Moreover, these did not include the viewpoint of psychosomatic and psychosocial survey [10].

In an effort to uncover the susceptibility and the causes of physical symptoms, psychiatric complaints and behavioral problems related to school children in Japan, we felt it would be beneficial and necessary to make a comparison on an international level using the same instrument. It was within practical reach for us to compare Japanese children with Swedish children; hence, our study was conceived.

As previously reported, we used a questionnaire including physical symptom and psychiatric complaint, self-esteem, coping style, life satisfaction and stressful life events. The study population comprised 742 Japanese children and 1120 Swedish children attending public compulsory schools grade 4-9 (10-15 years) [11].

**Figure 1:**
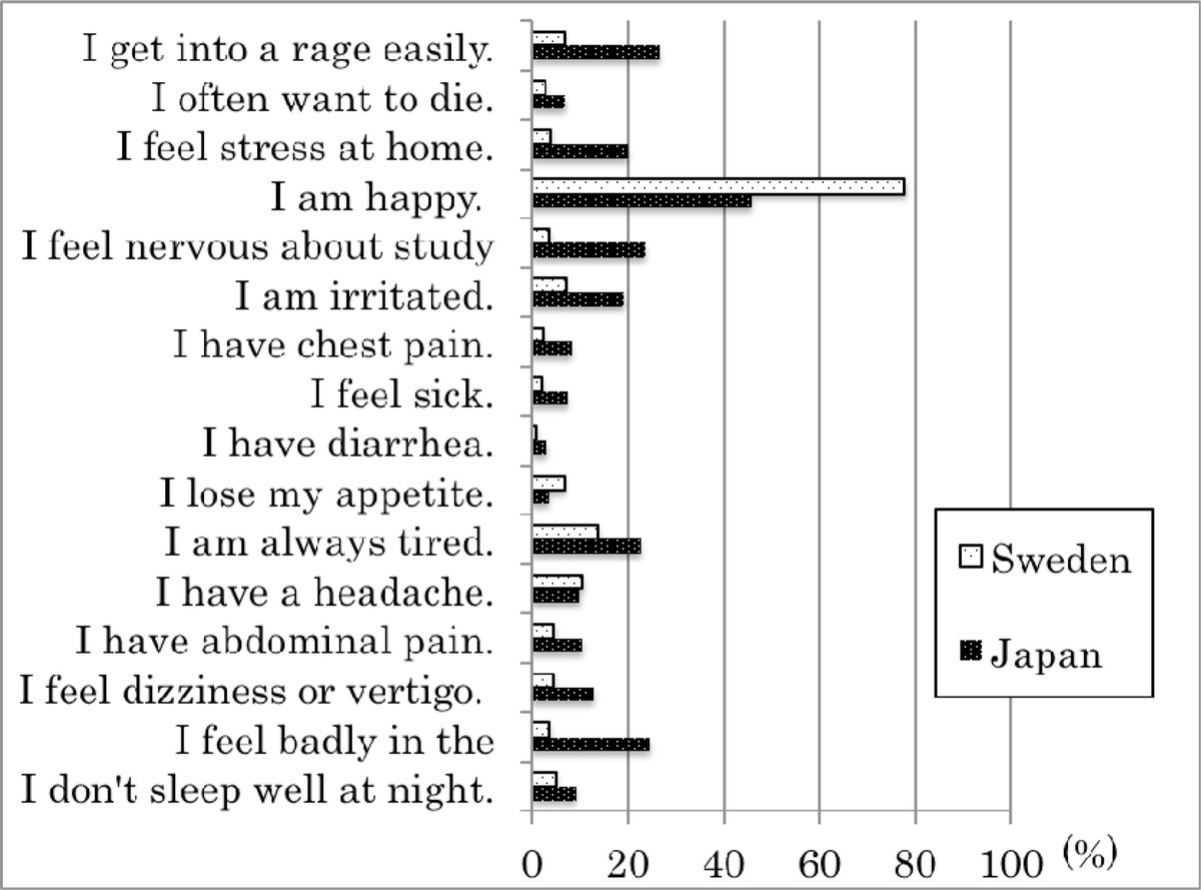
**Percentages of "yes" responses to physical symptoms and psychiatric complaints in Japanese and Swedish junior high school children**. All items that showed a significant difference between Japan and Sweden by Chi square analysis (p < 0.05).

As a result, the Japanese children had significantly higher physical symptom and psychiatric complaint scores than did the Swedish children as shown in Figure 1. Both the physical symptom and psychiatric complaint scores were significantly higher in adolescents than in preadolescents in Japan; this trend was less apparent in Swedish children. In addition, Japanese children were found to have considerably lower life satisfaction.

## Psychosocial background influencing psychosomatic symptoms in Japanese children, with special reference to school-related stress

As children's mental health problems seem to be attributable to stress and children spend more than approximately 15 000 hours in compulsory school, it appears to be valuable to reduce and to prevent school related stress. It has been suggested to arise from academic competition and interpersonal relations with classmates or teachers and surveys in the past clearly show that teachers' attitudes affect children's stress [12]. Samdal et al. found that satisfaction with school was correlated to their opportunities to influence regulations in school as well as feeling safe and having supportive teachers [13]. Also Resnick et al. found that if the students felt part of the school and were treated fairly by teachers, they had better emotional health and lower level of involvement in risky behavior [14].

**Figure 2:**
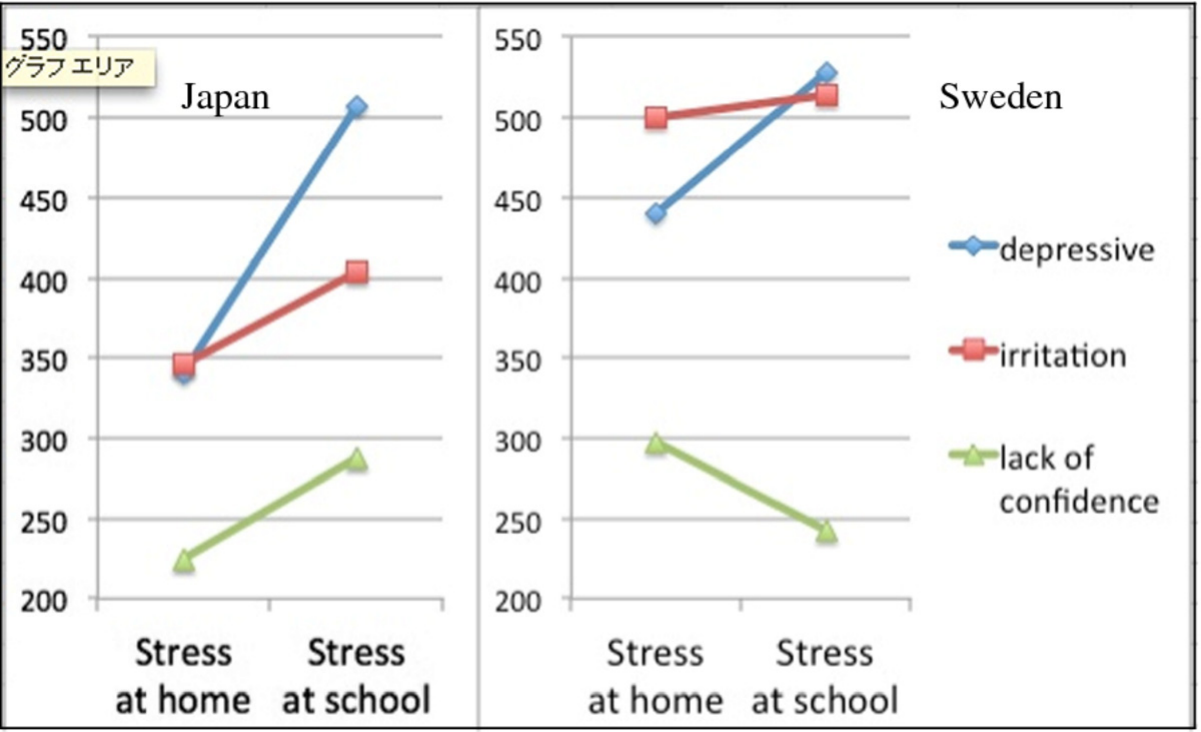
**Standardized multi-regression coefficient between psychological measures (depressive, irritation, lack of confidence) and emotional stress at home or that at school in junior high school pupils in Japan (left panel) and Sweden (right panel)**. The scale unit is indicated by 1/1000. Coefficients of measures of depressive, irritation and lack of confidence are higher for stress at school than for stress at home in Japanese children (left panel), although this trend is not apparent in Swedish children (right panel).

We have previously reported that physical symptoms and psychiatric complaints in children were significantly associated with school-related stressful events [15] on the basis of the survey conducted at Japanese elementary schools. We further investigated the influence of school-related stress or home-related stress on physical and psychological symptoms in Japanese adolescents in comparison with the Swedish counterparts. Psychological symptoms were divided into three sub-scales by factor analysis, ie, depressive tendency, irritation and lack of confidence, which were analyzed to determine the relation with the stress scale at school or at home using the multi-regression analysis. Obtained standardized multi-regression coefficients were shown in Figure 2. As a result, three subscales of psychological symptoms in Japanese children were more strongly influenced by school-related stress than by home-related stress. In Swedish children, on the other hand, the trend was not apparent.

In keeping with this, we found that the number of bullied and bullying children in Japan was double and six times, respectively, compared with Swedish children, indicating poor control of class management by the teachers and the delay of the legal system for preventing bullying at school.

These findings suggest that resolution of psychosocial problems in Japanese children should include comprehensive reformation of child health care systems in addition to educational systems; spread of bio-psycho-social pediatrics to general clinicians, promoting use of practical guidelines, training of specialists with standardized methods, in addition to psychosomatic education for school teachers.

## Enforcement of countermeasures for psychosomatic problems in Japanese children

The Japanese Society of Psychosomatic Pediatrics, a subcommittee of the Japan Pediatric Society, was founded in 1983 and will soon celebrate 30 years of establishment. The purpose of the establishment of the society is to promote clinical practices of psychosomatic medicine and to improve family and school circumstances in corporation with general pediatricians, psychologists, nurses, school teachers and other related personnel, and finally, to bring favorable mental/psychological development in children. The major activity of the society in the former period for thirty-years history included post-graduate trainings with serial special lectures for general pediatricians and publication of various researches. Nevertheless, in spite of our efforts, the number of children with psychosomatic diseases (OD, eating disorder, chronic headache, irritable bowel syndrome, recurrent abdominal pain in young children, recurrent vomiting, hyperventilation syndrome, bronchial asthma and incontinence) has continued rising.

In order to achieve the aim of the society, we have started several new projects to promote multi-center psychosomatic research and society-based activities since 1999 as follows.

1. to publish practical guidelines for child psychosomatic diseases: orthostatic dysregulation, eating disorders, chronic headache and abdominal pain and school absenteeism (2002-2006)

2. to start a grant-in-aid for participation in a special training program of epidemiology held by the national center (2003-4)

3. to make a list of domestic psychosomatic specialists and to set up the community-based medical network system [16] (2005-)

4. to establish local societies to promote community-based cooperation (2002-)

5. to establish the board certificate for specialists of child psychosomatic medicine (2010-)

We, hereby, shortly give summaries of the practical guideline published by the Society.

## Japanese clinical guidelines for child orthostatic dysregulation

This clinical practice guideline [17] provides recommendations for the assessment, diagnosis and treatment of school-aged children with orthostatic dysregulaiton (OD), usually named orthostatic intolerance in America and Europe. This guideline is intended for use by primary care clinicians working in primary care settings. The guideline contains the following recommendations for diagnosis of OD: 1) initial evaluation composed of including and excluding criteria, the assessment of no evidence of other disease including cardiac disease, etc.; 2) a new orthostatic test to determine four different subsets, ie, instantaneous orthostatic hypotension, postural tachycardia syndrome, neurally-mediated syncope and delayed orthostatic hypotension; 3) evaluation of the severity; and 4) judgment of psychosocial background with the use of rating scales. The guideline also contains the following recommendations for treatment of OD on the basis of the result of an orthostatic test in addition to psychosocial assessment; 1) Guidance and education to parents and children, 2) Non-pharmacological treatments, 3) Contact with school personnel, 4) The use of adorenoceptor stimulants and other medications, 5) Strategies of psychosocial intervention, 6) Psychotherapy. This clinical practice guideline is not intended as a sole source of guidance in the evaluation of children with OD. Rather, it is designed to assist primary care clinicians by providing a framework for decision making of diagnosis and treatments.

## Japanese clinical guidelines for chronic pain in children and adolescents

Chronic pain is a common problem in pediatric practice. The prevalence of chronic pain in children is more than 30%. Because pain indicates emotional expression as well as the physiological reaction toward infection, injury, and inflammation, both physiological and psychological assessments are essential to determine primary interventions for chronic pain. The Japanese Society of Psychosomatic Pediatrics Task Force of clinical practice guidelines for chronic pain in children and adolescents [18] compiled clinical evidence and opinions of specialists associated with primary care of pediatric chronic pain in "the Japanese clinical guidelines for chronic pain in children and adolescents" in 2009. The guidelines consist of three domains: "general introduction to chronic pain," "chronic pain in the lower abdomen," and "chronic headache." Each section contains information on the physiological mechanism, psychological aspects, assessment methods, and primary interventions for pediatric chronic pain. These guidelines are expected to help disseminate knowledge on primary interventions for chronic pain in children and adolescents.

## Japanese clinical guidelines for anorexia nervosa in childhood and adolescence

Basic lines of treatment of this guideline [19] include in the initial stage, (1) to perform physical treatment for malnutrition, to retain weight followed by gradual increase and to pay special attention to the refeeding syndrome, (2) psychologically to improve physical insight into disease and to reduce anxiety against weight gain under the daily diet. In the second stage, patients need to maintain the appropriate weight in normal daily life. Weight maintenance in parallel with more than 5 cm in height growth per year can be considered appropriate for children. The target of psychotherapy in the second stage is to let them realize that eating behavior was abnormal. In addition, counseling should be reinforced when psychological trauma or traumatic experiences is emerged. In the last stage, recovery of mental state to the normal level in addition to keeping the weight should be required along with elimination of psychological trauma. General pediatricians should be trained to treat patients in the initial stage and possibly in the second stage.

## Japanese clinical guidelines for school absenteeism

School absenteeism is the major problem in schools, especially in junior schools, as described above. This term is not a diagnostic criterion but represents the behavioral state as a consequence of the complex of physical, psychological, emotional, socio-ecological involvements. A typical school absenteeism goes through the following three periods, that is, (1) the early period is characterized by various physical complaints such as morning tiredness, headaches, abdominal pain, dizziness, etc., followed by intermittent absence from school or being late for school, (2) the resting period shows the withdrawal at home, loosing motivation, and sometimes depressive or sometimes irritative, and domestic violence arises, (3) the recovery period, about 70% of school absentees at age 15 have been reported to come back to school, or to start working. There is still controversial regarding medical care for school absenteeism, although pediatricians should treat them physically and mentally from the standpoint of promoting normal development of children. The following advises [20] are useful.(1) Physical symptoms are sometimes caused by commorbid diseases including orthostatic dysregulation, irritable bowel syndrome, migraine, and other autonomic dysfunction which are often affected by emotional stress.(2) Emotional stress includes family problems (parent rearing, maltreatment), school affairs (bullying, academic performance, teacher's management etc.).(3) A longstanding follow-up treatment is needed including psychological support by co-medical staffs (psychologists, medical social workers, school workers) and use of other social resources to help children and parents.

## Competing interests

The authors declare that they have no competing interests.

## Authors' contributions

HT and ST made the questionnaire inventory and carried out it in Japan, MB did it so in Sweden, ST analyzed with statistical method, and OT arranged and unified the research. All authors read and approved the final manuscript.

## Ethical approval

This study was approved by the ethical committee on 28 March 1995, in Osaka Medical College and Gothenberg University.
